# Diagnostic accuracy of procalcitonin for overall and complicated acute appendicitis in children: a meta-analysis

**DOI:** 10.1186/s13052-019-0673-3

**Published:** 2019-07-09

**Authors:** Wei Cui, Haipeng Liu, Hong Ni, Xianhui Qin, Liran Zhu

**Affiliations:** 1 Department of Scientific Research and Education, Anhui Provincial Children’s Hospital, Wangjiang Road, Hefei, 230051 Anhui China; 2 Anhui Institute of Pediatric Research, Hefei, Anhui China; 3 National Clinical Research Center for Kidney Disease; State Key Laboratory for Organ Failure Research; Renal Division, Nanfang Hospital, Southern Medical University, Guangzhou, China

**Keywords:** Acute appendicitis, Diagnosis, Meta-analysis, Pediatric, Procalcitonin

## Abstract

**Background:**

Diagnostic value of procalcitonin (PCT) for acute appendicitis (AA) has been evaluated in adult patients, but the application in children remains controversial. The aim of this study was to evaluate the diagnostic value of PCT for overall and complicated AA in children.

**Methods:**

The PubMed, EMBASE, Web of Science, Cochrane Database of Systematic Reviews, Chinese National Knowledge Infrastructure, and Wanfang were searched along with reference lists of relevant articles up to January 2018 without language restrictions. Original articles that reported the performance of PCT in the diagnosis of pediatric AA and associated complications were selected. To assess the diagnostic value of PCT, sensitivity, specificity, diagnostic odds ratios (DORs), summary receiver operating characteristic (ROC) curves, area under the curve (AUC), and 95% confidence intervals (95% CIs) were estimated.

**Results:**

Seven qualifying studies (504 confirmed AA and 368 controls) from 6 countries for overall AA and 4 studies (187 complicated AA and 185 uncomplicated AA) for complicated AA from 3 countries were identified. The pooled sensitivity and specificity of PCT for the diagnosis of pediatric AA were 0.62 (95% CI: 0.57–0.66) and 0.86 (95% CI: 0.82–0.89), respectively. The DOR was 21.4 (95% CI: 3.64–126.1) and the AUC was 0.955. PCT was more accurate in diagnosing complicated appendicitis, with a pooled sensitivity of 0.89 (95% CI: 0.84–0.93), specificity of 0.90 (95% CI: 0.86–0.94), and DOR of 76.73 (95% CI: 21.6–272.9).

**Conclusion:**

This meta-analysis showed that PCT may have potential value in diagnosing pediatric AA. Moreover, PCT had greater diagnostic value in identifying pediatric complicated appendicitis.

## Background

Acute appendicitis| (AA) is the most frequent abdominal surgical emergency in children [1]. Although the mortality rate of pediatric AA has declined in recent years, delay in diagnosis and treatment for AA result in an increased perforation rate, post-operative morbidity, mortality, and hospital length of stay [2]. Approximately 16.5% of appendicitis progresses to complicated appendicitis, such as perforation, gangrene, peritonitis, or abscess formation, which may lead to higher costs and even death [3, 4]. Moreover, patients with complicated AA are more likely to have post-surgical complications (abscesses, intestinal obstruction, and hemodynamic instability) [5] than patients with uncomplicated AA. It is crucial to identify AA early and to discriminate uncomplicated and complicated AA so that preemptive therapy can be performed.

Non-specific signs (irritability, anorexia, and lethargy) in patients with appendicitis are difficult for parents and pediatricians to interpret, thus leading to a high misdiagnosis rate. Overall, the misdiagnosis rates of AA are 70–100% in children < 3 years of age, 19–57% in preschool children, 12–28% in school-aged children, and up to 15% in adolescents [6]. In-hospital observation and repeated clinical examinations during evaluation of a patient with AA may easily lead to an increase in false-positive diagnoses and operations [2]. The use of ultrasound, computerized tomography (CT), and magnetic resonance image (MRI) could potentially improve the accuracy of AA; however, use remains limited due to radiation exposure risk, lifetime risk of cancer, the increase in cost, and lack of universal availability [7, 8]. Recently, biomarkers, such as procalcitonin (PCT), have been shown to have potentially good diagnostic accuracy and reliability, which may be more appropriate indices in the diagnosis of appendicitis, and in some cases predict the severity of the condition.

PCT is the prohormone of calcitonin. PCT levels rise rapidly after systemic bacterial infection but remain low in viral infections and inflammatory diseases. The role of PCT in the diagnosis of pediatric AA is still inconclusive. The aim of the current meta-analysis was to evaluate the diagnostic value of PCT for overall and complicated AA in children.

## Material and methods

### Search strategy and selection criteria

We searched PubMed, EMBASE, CNKI, and Wanfang from inception to April 2018 with MeSH terms (“procalcitonin OR procalcitonin OR PCT” and “appendicitis OR ecphyaditis OR epityphlitis OR acute appendicitis or chronic appendicitis” and “pediatric OR paediatric OR child OR children”). Additionally, manual searches were performed of bibliographies for all relevant trials and review articles. The searches were restricted to human studies and clinical trials. There were no language restrictions. A team of experts in the relevant field was assembled.

A standard protocol for study selection and data abstraction was developed by our multidisciplinary team. Studies that met the following criteria were included: (1) evaluation of PCT to diagnose pediatric AA and associated complications; and (2) sufficient data to construct a 2 × 2 contingency table. The exclusion criteria included: (1) a lack of PCT diagnostic accuracy; (2) insufficient data to reconstruct 2 × 2 tables; and (3) case reports, review articles, editorials, and clinical guidelines.

### Data extraction

All data from eligible studies were independently abstracted in duplicate by two investigators according to standard protocol and reviewed by a third investigator. Discrepancies were resolved through discussion with the multidisciplinary research team that developed the protocol. The following data were extracted: first author’s name; year of publication; study population characteristics; number of patients and controls included; methods for PCT measurement; cut-off values; and results of TP, true positive; FP, false positive; TN, true negative; FN, false negative.

### Quality assessment

The quality of the selected studies was assessed by means of the Quality Assessment of Diagnostic Accuracy Studies (QUADAS)-2 criteria [9], which consists of the following four domains: patient selection; index test; reference standard; and flow and timing. The inconsistency index (*I*^*2*^) was calculated to quantify the extent of heterogeneity. In this analysis, the pathologic examination of the surgical specimen was used as the reference standard to verify AA for patients undergoing surgery. We have reported according to Preferred Reporting Items for Systematic Reviews and Meta-Analyses-2 (PRISMA-2) and Assessing the Methodological Quality of Systematic Reviews (AMSTAR) guidelines.

### Statistical analysis

The pooled sensitivity, specificity, positive likelihood ratio (PLR), negative likelihood ratio (NLR), and diagnostic odds ratios (DORs) of PCT were calculated for each included study. A summary ROC curve was created by plotting the true-positive rate (sensitivity) against the false-positive rate (1 – specificity) at various cut-off values from individual studies using random-effect models. The threshold effect was investigated using the Spearman correlation coefficient. Statistical analyses were performed using Meta-Disc 1.4 for Windows and Review manager 5.3. All statistical tests were two-sided with statistical significance set at *P* < 0.050.

## Results

**Fig. 1:**
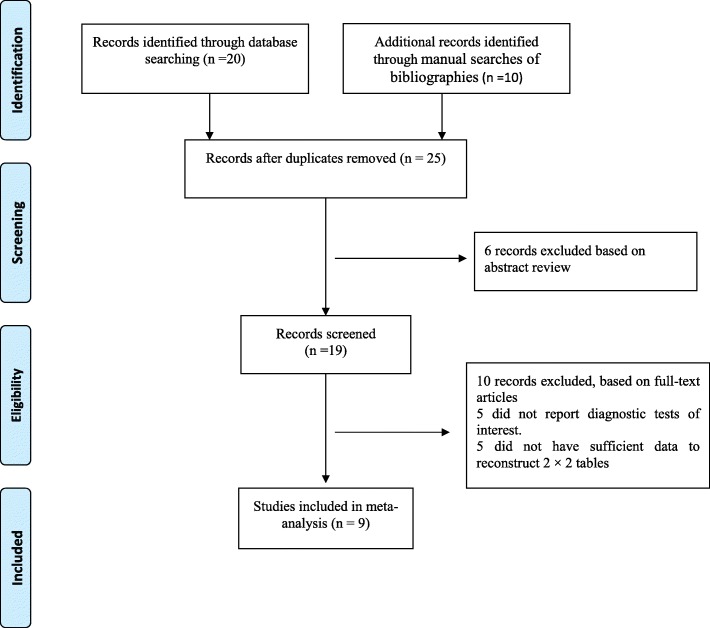
PRISMA flowchart of study selection

Twenty-five potentially relevant papers were retrieved using the systematic search. Our initial literature search identified 15 studies for detailed assessment. Of these studies, four were excluded due to a lack of data on the diagnostic tests of interest and two did not have sufficient data to reconstruct 2 × 2 tables (Fig. 1). Our final analysis included nine studies [5, 10–17]. The identified studies, published between 2005 and 2017, were conducted in 6 countries (India, Greece, France, Spain, China, and Turkey) with sample sizes ranging from 50 to 212 participants. Seven studies [10, 11, 13–17] reported the use of PCT in the diagnosis of overall appendicitis and four studies [5, 12, 15, 16] provided the predictive value of PCT for complicated AA in pediatric patients. Tables 1 and 2 summarize the characteristics of the included studies that used PCT to assess overall and complicated appendicitis.

**Table 1:** Characteristics of the included studies that used PCT to assess pediatric acute appendicitis in the meta-analysis

First author	Year of publication	Country	AA	Control	Age, years;Mean (range)	PCT testing system	Cut-off (ng/ml)	Sensitivity (%)	Specificity (%)	No. of TP	No. of FP	No. of FN	No. of TN
Alkan [10]	2017	Turkey	56	40	9 (7–11)	LUMI test	0.37	32.3	89.7	18	4	38	36
Benito [17]	2016	Spain	89	96	9.32 ± 2.7	LUMI test	0.1	38.1	64.5	34	34	55	62
Wang [11]	2016	China	96	56	7.4 ± 2.1	LUMI test	0.5	85.4	90.6	82	5	14	51
Li [13]	2015	China	42	36	12(5–17)	LUMI test	0.5	95.2	88.9	40	4	2	32
Chandel [16]	2011	India	23	27	3–15	LUMI test	0.5	95·6	100	22	0	1	17
Kafetzis [15]	2005	Greece	130	82	9 ± 2.2	LUMI test	0.5	73·0	95·0	47	8	7	140
Kouame [14]	2005	France	68	31	2–15	LUMI test	0.5	28·0	100	19	4	49	29

Abbreviations: *PCT* procalcitonin, *AA* acute appendicitis, *TP* true positive, *FP* false positive, *TN* true negative, *FN* false negative, *LUMItest* immunoluminometric method

**Table 2:** Characteristics of the four included studies that used PCT to assess pediatric complicated appendicitis

First Author	Year of Publication	Country	AA	CA	Age, years:Mean (range)	Cut-off (ng/ml)	Sensitivity (%)	Specificity (%)	No. of TP	No. of FP	No. of FN	No. of TN
Yuchi [12]	2015	China	38	70	15.2 ± 2.1	0.5	81	82	57	7	13	31
Teresa [5]	2012	Spain	69	42	1.2–17.1	0.18	97	80	67	8	2	34
Chandel [16]	2011	India	12	11	3–15	0.74	100	100	11	0	0	12
Kafetzis [15]	2005	Greece	66	64	9 ± 2.2	0.5	73.0	95.0	47	2	17	64

Abbreviations: *PCT* procalcitonin, *AA* acute appendicitis, *CA* complicated appendicitis, *TP* true positive, *FP* false positive, *TN* true negative, *FN* false negative

**Fig. 2:**
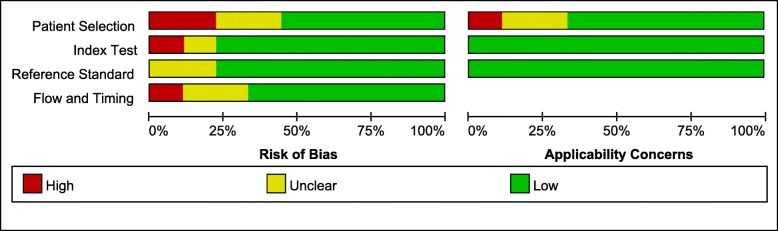
Graph displaying the percentage of studies with varying degree of bias and varying applicability for each of the four QUADAS-2 domains

Quality assessment was based on the QUADAS-2 guidelines for all nine studies, as shown in Fig. 2. Four studies had an ‘unclear’ or ‘high’ risk of bias concerning patient selection due to inconsistent inclusion criteria. Only two studies had an ‘unclear’ or ‘high’ risk of bias with respect to the index test and reference standards due to the same reference standard (histologic examination) and the near-same thresholds in which the studies were assessed. Three studies had an ‘unclear’ or ‘high’ risk of bias with respect to patient flow.

### Diagnostic accuracy analyses

**Fig. 3:**
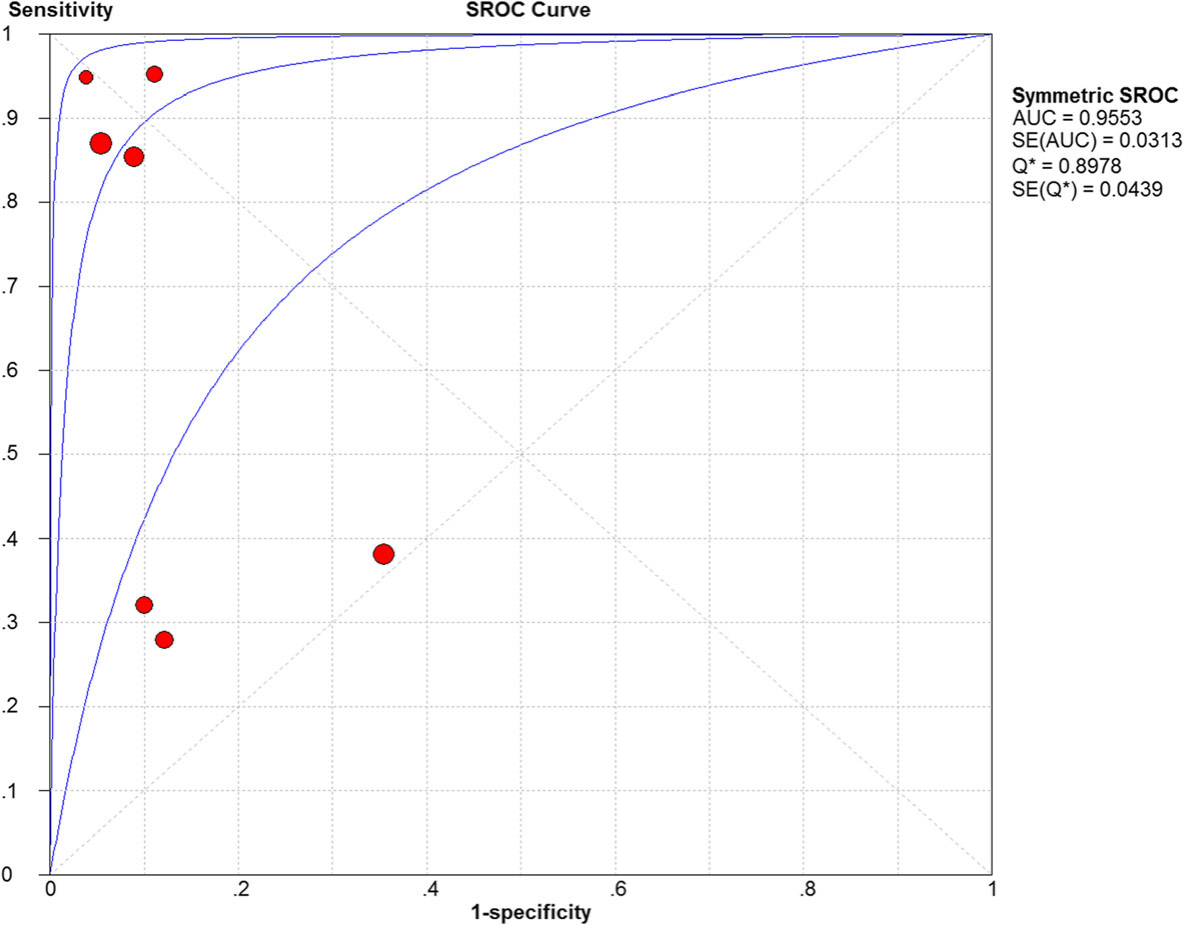
Receiver operating characteristic (ROC) curve analysis for the diagnosis of children with acute appendicitis using a procalcitonin, AUC = area under the curve, SROC = summary receiver operating characteristic

**Fig. 4:**
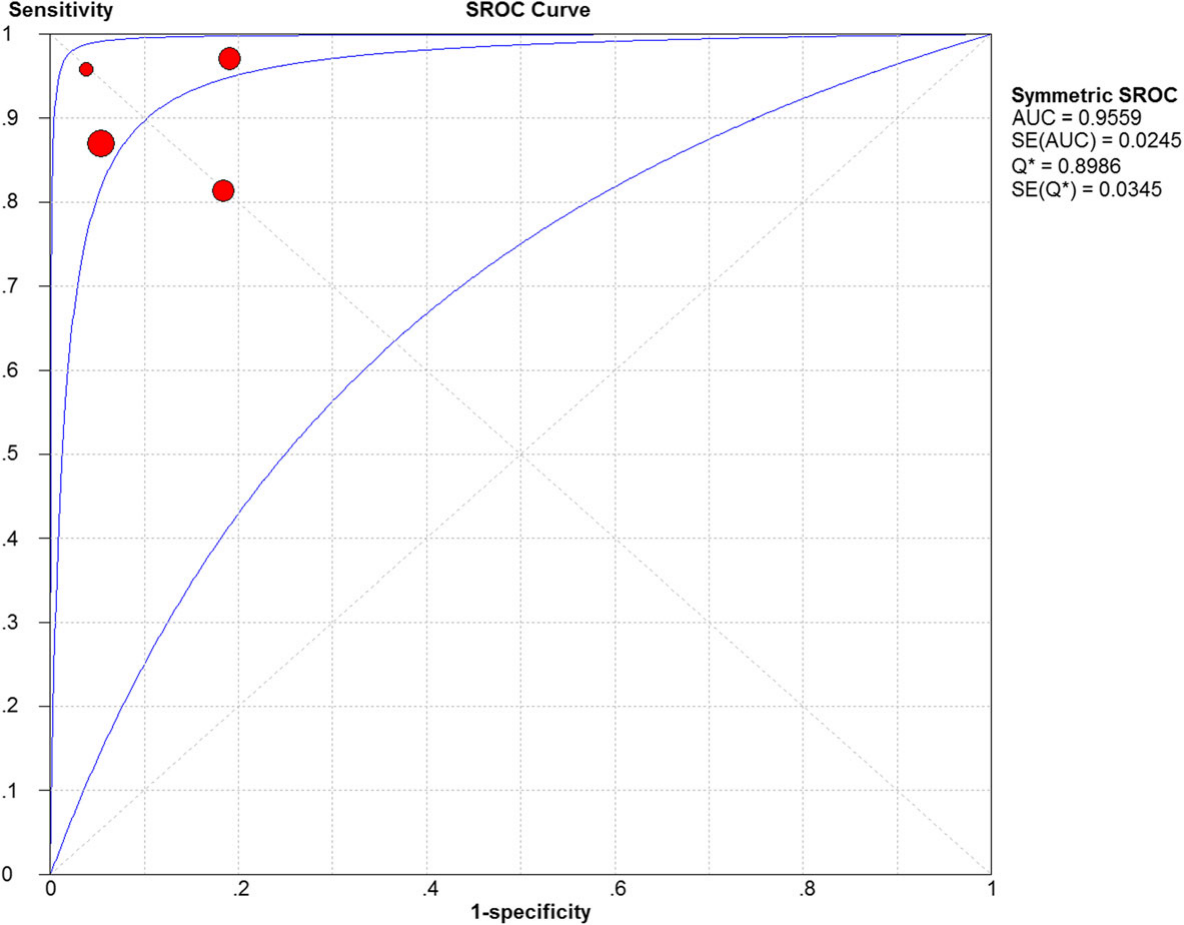
Receiver operating characteristic (ROC) curve analysis for the prediction of children with complicated appendicitis using a procalcitonin, AUC = area under the curve, SROC = summary receiver operating characteristic

The Spearman correction coefficient between PCT and pediatric AA was calculated to be 0.5 (*P* = 0.253), indicating that there is no heterogeneity from threshold effects. The pooled DOR for overall and complicated AA was 21.4 (95% confidence interval (CI): 3.64–126.1) and 76.7 (95% CI: 21.57–272.9), respectively. The AUC for the summary ROC of the PCT test for diagnosis of overall AA was 0.955 followed by 0.956 for complicated AA, indicating a higher predictive value for complicated AA in children, as shown in Figs. 3 and 4.

### Overall diagnostic indices

The pooled sensitivity and specificity for PCT are shown in Table 3. Overall, the meta-analysis showed a pooled sensitivity of PCT for the diagnosis of overall AA of 0.62 (95% CI: 0.57–0.66; *I*^*2*^ = 96.3%) and specificity of 0.86 (95% CI: 0.82–0.89; *I*^*2*^ = 86.7%). The combined PLR was 5.33 (95% CI: 1.79–15.8) with respect to the combined NLR of 0.29 (95% CI: 0.15–0.58). The pooled sensitivity and specificity of PCT for the diagnosis of complicated AA were 0.89 (95% CI: 0.84–0.93; *I*^*2*^ = 76.1%) and 0.90 (95% CI: 0.86–0.94; *I*^*2*^ = 75.3%), respectively. The combined PLR was 7.68 (95% CI: 3.66–16.14) with respect to the combined NLR of 0.12 (95% CI: 0.05–0.27).

**Table 3:** Summary of diagnostic accuracy parameters for meta-analysis

	No. of studies	Sensitivity (%)	Specificity (%)	Positive likelihood ratio	Negative likelihood ratio	Diagnostic OR	Area under ROC curve
PCT for AA	7	62(57,66)	86(82,89)	5.33(1.79,15.84)	0.29(0.15,0.58)	21.4(3.64,126.1)	0.955
PCT for CA	4	89(84,93)	90(86,94)	7.68(3.66,16.14)	0.12(0.05,0.27)	76.7(21.6, 272.9)	0.956
PCT for Asia people	3	90(84,94)	91(84,96)	9.57(5.21,17.55)	0.10(0.04,0.23)	89.0 (36.7,215.9)	0.963
PCT for non-Asia people	4	44(38,50)	84(80,88)	3.35(0.80,14.1)	0.60(0.36,0.99)	6.21(0.77,50.2)	0.899

Abbreviations: *PCT* procalcitonin, *AA* acute appendicitis, *CA* complicated appendicitis

### Subgroup analysis of source populations

The results of subgroup analysis are summarized in Table 3. Analysis of three studies from Asian populations had a superior diagnostic accuracy for PCT with pooled sensitivity of 0.90 (95% CI: 0.84–0.94, *I*^*2*^ = 61.6%), specificity of 0.91 (95% CI: 0.84–0.96; *I*^*2*^ = 17.8%), and an AUC of 0.963 compared with the overall pooled estimates (sensitivity of 0.62 (95% CI: 0.57–0.66) and specificity of 0.86 (95% CI: 0.82–0.89)). The corresponding values for the source populations from non- Asian populations were 0.44 (95% CI: 0.38–0.50) for sensitivity, 0.84 (95% CI: 0.80–0.88) for specificity, and 0.90 for the AUC (Table 3).

## Discussions

PCT is the prohormone of calcitonin, with normal plasma reference levels of 0.1–0.5 ng/ml in healthy individuals. The PCT level increases rapidly after a systemic bacterial infection, but with no significant increase in viral infections, making PCT one of the most important early laboratory signs for systemic bacterial and fungal infections [18–20]. Several studies have shown PCT to be an excellent marker of bacterial infections and the PCT level increases rapidly with the severity of infection in children [21, 22]. As appendicitis is difficult to be diagnosed in a timely fashion, which delays surgical intervention, it is meaningful to evaluate the value of PCT in diagnosing AA and complicated AA, as in the present analysis.

Our study comprehensively evaluated the diagnostic value of PCT in pediatric patients with AA with a sensitivity of 0.62 (95% CI: 0.57–0.66) and a specificity of 0.86 (95% CI: 0.82–0.89), which indicated the potential value in diagnosing pediatric AA. Also, the mean value of PCT was significantly higher in the appendicitis group in these studies under review. Of note, a higher sensitivity and specificity were observed in pediatric patients with complicated AA (sensitivity, 0.89 (95% CI: 0.84–0.93) and specificity, 0.90 (95% CI: 0.86–0.94)) than the overall AA. Hence, our study suggests a better diagnostic accuracy for PCT in complicated AA than overall AA.

Previous research findings on the diagnostic value of PCT in AA and complicated AA have been inconsistent [2, 23, 24]. Yu et al. [23] showed a low value of PCT in diagnosing AA with a pooled sensitivity of 0.33 and a specificity of 0.89 in patients with all ages, including adults and children; however, Wu et al. [24] observed a useful application with complicated appendicitis than in overall AA in adults. Moreover, a study by Muhammad et al. [25] found that PCT were applicable in diagnosing AA with sensitivity and specificity of 0.85 and 0.74 in an adult population, which outperformed serum CRP. The possible reasons for these contradictory findings include differences in study methodology, sample size, and population. Our subgroup analysis also suggested high levels of sensitivity and specificity in Asian children (India and China), which indicated possible population-based diagnostic effects; however, the exact mechanisms regarding the diagnostic value of PCT to AA in each population warrants further investigation, and our findings should also be confirmed.

The diagnostic value of other biomarkers, such as the C-reactive protein (CRP), white blood cell (WBC) count, bilirubin, and interleukin 6 (IL-6) levels for AA have been evaluated in literatures [26–33]. For example, Beltran et al. [27] accessed high sensitivity for WBC counts and CRP levels (sensitivity, 0.9–1.0) to differentiate between patients with and without appendicitis, but low specificity (0.2–0.4) was observed. Gürleyik et al. [26] found that serum IL-6 measurement is not of benefit in increasing the accuracy of the diagnosis of appendicitis with unacceptably high false-negative and -positive rates (16 and 54%, respectively). Bilirubin was reported to have the highest sum of sensitivity (0.61) and specificity (0.61) with a threshold of 15 μmol/L by Kaser et al. [30]. However, there were insufficient evidence that proved the prior diagnostic accuracy and reliability than PCT. Of note, the overall sensitivity (0.62), specificity (0.86), and PLR (5.33) for PCT indicated a potential predictor for AA but not sufficient as a stand-alone measure to rule in or rule out AA, thus we suggest the possibility of utilizing PCT, clinical symptoms, and radiation (ultrasound, CT, and MRI) in an effort to increase the diagnostic accuracy in children with AA.

The present meta-analysis had some limitations. First, due to a focusing on pediatric patients, the included studies were limited in number. Also, some studies that reported both adults and children, but did not construct diagnostic data in children were excluded. Therefore, few studies with small sample sizes were included in this meta-analysis compared with adult analyses. Second, with no pre-existing widely accepted threshold, various diagnostic cut-off values were used in complicated AA; however, no heterogeneity form threshold effects were observed. Third, some of the included studies exhibited bias. Fourth, considering the cost of PCT detection, the benefit-to-cost ratio remains controversial. Therefore, our study was just hypothesis-generating. All findings need to be further investigated and confirmed in future studies.

## Conclusions

In conclusion, our study suggests that PCT has potential diagnostic value in overall pediatric AA and has greater diagnostic value in identifying complicated pediatric AA. More studies are needed to further investigate and confirm the results.

## Data Availability

All data generated or analyzed during this study are included in this published article.

## Abbreviations

AA: Acute Appendicitis
AUC: Area Under the Curve
CI: Confidence interval
DOR: Diagnostic odds ratios
FN: False negative
FP: False positive
PCT: Procalcitonin
ROC: Receiver operating characteristic
TN: True negative
TP: True positive
